# Climate-Driven Evolution

**DOI:** 10.1371/journal.pbio.1000383

**Published:** 2010-06-08

**Authors:** Henry Harpending

**Affiliations:** Department of Anthropology, University of Utah, Salt Lake City, Utah, United States of America

**Figure:**
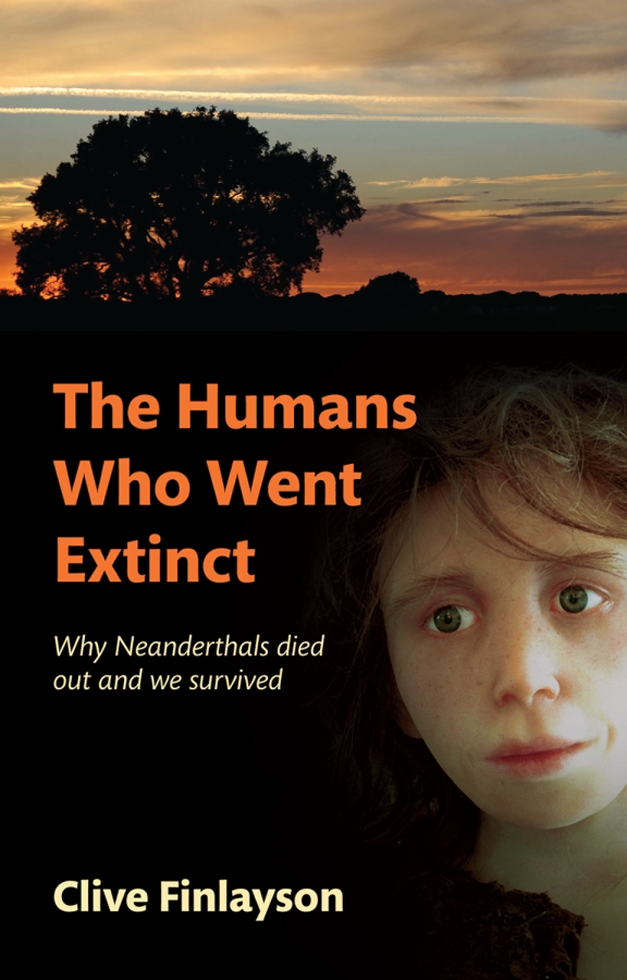
Finlayson C (2009) The Humans Who Went Extinct: Why Neanderthals died out and we survived. New York: Oxford University Press. pp. XI+273. ISBN (cloth): 978-0-19-923918-4. US$29.95.

There are several rather distinct traditions in the contemporary study of human evolution. One is the detailed study of anatomical differences among fossils. A second emphasizes classical ecology with detailed attention to ancient climates and environments and comparative biogeography. A third has a focus on behavioral ecology (i.e., what used to be called sociobiology). It appears to this outsider that these are not so much competing traditions as they are nearly independent and seemingly unaware of each other. They each have much to contribute, they are each prominent in particular current disagreements in the field, and they each have glaring weaknesses.

Anatomical details are central to the literature on Australopithecines and their relatives, primates in Africa on the human side of the ancient chimp–human split from five to ten million years ago to about two and half million years ago when we start calling the fossils *Homo* rather than *Australopithecus*. The anatomists are also in the thick of debates these days about the hobbit, the small hominid remains found on the island of Flores. Details of the wrist are apparently critically important in distinguishing whether these creatures are derived from Australopithecines, from *Homo erectus* (perhaps having undergone island dwarfism), or are simply pathological dwarves. A weakness of the anatomical tradition is the treatment of anatomical details as if they were selectively neutral, mere traits that can be tabulated to generate answers by consensus. There is not much awareness of quantitative genetics.

The behavioral ecology tradition emphasizes mating competition and intergroup struggles as the important drivers of human evolution. With roots in economics, comparative animal behavior, and the new population genetics of interaction started by W.D. Hamilton in the 1960s, social interactions themselves are the prime drivers of human evolution. For decades there has been a kind of faith that there was some “environment of evolutionary adaptedness” (EEA) in the past to which humans are well adapted and that if we could know about this EEA then we could understand human nature.

The ecologists look to ancient environments, climate, and climate change to explain changes in the distribution of humans and related species in time and space. They see humans responding to the physical and biotic factors like geography, climatic shifts, the availability of exploitive technologies, or the availability of domesticates. In this literature, humans are members of communities, and these communities are like organisms evolving to become better and better at provisioning their members. This group or species view of evolution contrasts sharply with the implicit model of the behavioral ecologists in which reproductive competition between individuals within groups is the important dynamic.

*The Humans Who Went Extinct* is firmly in the third, that is, classical ecological tradition. This admirable book is a summary and interpretation of human evolution from the first bipeds several million years ago to the earliest settled agricultural populations after the end of the Pleistocene. The title does not do justice to the scope of the book: there is only a weak focus on the extinction of the Neanderthals. It is an excellent summary of the classical ecological approach to human evolution with a dazzling portrayal of the relevant paleoclimatology, paleoecology, and biogeography.

These traditions ought to be complementary; instead they are rather isolated from each other. The discipline would be enriched if we acknowledged that the narratives that each tells are models and that models should be evaluated and falsified with data. There are numerous situations where models from the ecological and behavioral traditions compete directly, and such situations ought to be exploited for direct tests of the competing models.

Why did settled farming appear after the Last Glacial Maximum in several areas of the world, but never in the prior interglacial about 120,000 years before? The ecologists explain this in terms of geography and climate, the availability of domesticates, increasing demand from a larger human population, and a lot of chance. Some in the behavioral ecology tradition see settled agriculture as a consequence of the formation of regional polities with militias that suppress local raiding and warfare, leading to population growth, land scarcity, and increasing labor inputs into subsistence. These models could both be true or both false, but they ought to be tested as competing hypotheses.

Another playground of competing explanations in extant narratives is the “creative explosion” in Europe and Western Asia about 35,000 years ago. There appear quite suddenly in the archaeological record new ways of making stone tools, worked bone, decorations like beads, evidence of clothing, and art including sculptures like the famous Venus figurines. One explanation is that some cognitive or perhaps social threshold was crossed marking the origins of true humanness. The creativity, the evidence of regional exchange of materials, the art and fine technology—all these mark here the very first real members of our species. A second, perhaps more cynical, explanation is that whenever we see gaudy art, fine weapons, and fancy technology among technologically primitive societies today, these traits mark societies where men don't have to work very hard provisioning their families. Think of the societies of the US Northwest, where salmon runs only take up several months per year, with the rest of the caloric input from berries and gathered foods presumably gathered mostly by women. The result is beautiful totem poles and watercraft and other decorative arts. Another familiar example is the tribes of the US Great Plains after they took up mounted predation of bison following the introduction of the horse. A small hunt could feed many people for weeks, leaving males free to develop an impressive warrior culture with lots of gaudy decorations and weapons. In the view of cynics, then, the creative explosion is a mere signature of a society where men could parasitize women. The makers of the Venus figures are not foreshadowing Rodin, they are foreshadowing *Playboy* magazine. Can these models be tested?

I am no expert in the classical ecological tradition; so much of Finlayson's fascinating book was new to me. For example, Finlayson calls on his knowledge of biogeography to propose that Australopithecines, like azure winged magpies, occupied a wide belt across Central to Eastern Asia in an ecosystem in which they flourished. This proposal is not in our standard textbooks, but the great paleontologist GHR von Koenigswald always maintained that he found many Australopithecine teeth in his collections from Chinese pharmacists.

A staple of my lecture notes on the modern human diaspora is that when the earth is cold the Middle East is ecologically Europe with its Palearctic fauna, while warming means the return of Africa fauna to the Middle East. Several modern looking skulls from the Middle East (Skuhl, Qafzeh) then simply represent a temporary intrusion of African fauna into the Levant ca. 120 kya. Finlayson destroys this understanding of mine, pointing out that the faunal associations of the early moderns in the Levant, and of the slightly later Neanderthals, were much the same.

Another staple of my lectures is that the appearance of anatomically modern humans in Europe is marked by a new technology called Aurignacian (distinguished by the use of blade tools) brought by the moderns. Finlayson casually destroys this staple too, pointing out that there is no known association between the Aurignacian and modern human remains. The Aurignacian could very well have been made by Neanderthals.

Even worse, I repeat the standard narrative that moderns out-competed Neanderthals and were somehow responsible for their extinction. Not so fast, says Finlayson, suggesting that climate change drove the Neanderthals away and they may never have even encountered the modern human invaders. His candidate for an unequivocal archaeological marker of moderns is the Gravettian cultural tradition with its origin in western Asia, a tradition that was able to exploit open country herd prey on the expanding tundra.

This book is packed with new data, insights, and perspectives, with the dominant theme being about how climate has driven human evolution. The narrative starts with the handful of very early apparent precursors of the Australopithecines and the later appearance of *Homo erectus* with its larger brain and slightly advanced tool-making technology. The conventional story is that this new version of *Homo* evolved in Africa and left about one and one half million years ago, but Finlayson points out that there is plenty of room for a hypothesis of an Asian origin of this taxon. Several chapters discuss the earliest modern humans in Europe and in Southeast Asia and Oceania, again with a narrative emphasizing climate change and shifting ecological zones as drivers of the process. The discussion of the terrifying climatic instabilities of the Pleistocene, particularly in western Eurasia, is particularly valuable. The appearance of sedentary communities soon after the end of the Pleistocene occurred well before the appearance of agriculture, contradicting our established understanding that agriculture led to sedentary lifestyles. The spectacular site of Göbekli Tepe in Southwestern Anatolia with its monumental architecture is a particularly dramatic example. Finlayson points out that the rich foragers of the American Northwest Coast were likely on the same path evolutionary path.

The book is wordy and repetitious in places. It would have benefitted from the heavy hand of an editor in several ways: it would be shorter and more concise and adequate space would have been given to the maps, which are obviously important and informative but which are printed at such a scale that they are nearly impossible to interpret. The author commands a dense set of information about ancient climates and geochronology, but readers unfamiliar with the information will have a difficult time following the narrative in many places. Again, a heavy-handed editor might have insisted on more timelines and diagrams in the text.

The book is somewhat dense for an introductory course, but it would be just right for an advanced course on human evolution. An ideal advanced course might use Klein [1], an encyclopedic yet accessible survey including the anatomical details of the fossil record, the volume under review for the classical ecological approach, and Boyd and Silk [2] for the behavioral ecology.

As a population geneticist, I must explain why I have not discussed genetics in this review. In the heady times of mitochondrial DNA in the 1980s it seemed that genetics would answer important questions about human evolution. Since then, we seem to know less and less: the mutation rate of mtDNA slows down as larger time intervals are considered, and it has become clear that haploid markers like mtDNA and the Y chromosome are not very reliable markers of origins and ancestry. The most important finding about our genetics from the nuclear genome was the apparent small effective size of our species, on the order of 10,000 individuals. We knew in the 1980s that this was due to some severe bottleneck or bottlenecks in human history, but since then it has become apparent that other demographic phenomena, like waves of advance and selective sweeps of advantages genes and haplotypes, could generate the apparent small effective size. In other words, we know much less today from genetics than we thought we did two decades ago.
